# Antibacterial Effects of Afzelin Isolated from *Cornus macrophylla* on *Pseudomonas aeruginosa*, A Leading Cause of Illness in Immunocompromised Individuals

**DOI:** 10.3390/molecules19033173

**Published:** 2014-03-17

**Authors:** Sang Yeol Lee, Young-Jin So, Moon Sam Shin, Jae Youl Cho, Jongsung Lee

**Affiliations:** 1 Department of Dermatological Health Management, College of Health Science, Eulji University, Seongna-Si, 461-713 Gyeonggi-Do, Korea; E-Mails: yjso@eulji.ac.kr (Y.-J.S.); msshin@eulji.ac.kr (M.S.S.); 2 Department of Life Science, Gachon University, San 65, Bokjeong-Dong, Sujeong-Gu, Seongnam-Si, 461-701 Gyeonggi-Do, Korea; E-Mail: leesaye@gachon.ac.kr; 3 Department of Genetic Engineering, Sungkyunkwan University, Suwon-Si, 440-746 Gyeonggi-Do, Korea; 4 Department of Senior Healthcare, BK21 plus program, Graduated school, Eulji University, Seongna-Si, 461-713 Gyeonggi-Do, Korea

**Keywords:** *Cornus macrophylla*, antibacterial activity, kaempferol 3-*O*-α-L-rhamnopyranoside, *Pseudomonas aeruginosa*

## Abstract

The crude ethyl acetate extract of the leaves of *Cornus macrophylla* showed antibacterial activity against *Pseudomonas aeruginosa*, a leading cause of illness in immunocompromised individuals. Bioactivity-guided separation led to the isolation of kaempferol 3-*O*-α-L-rhamnopyranoside (afzelin). The structure was determined based on evaluation of its spectroscopic (UV, MS, and NMR) data. The minimum inhibitory concentration (MIC) of afzelin against *Pseudomonas aeruginosa* was found to be 31 µg/mL. In addition, the results indicated that a hydroxyl group at C3 of the C-ring of the flavone skeleton and the rhamnose group may act as a negative factor and an enhancing factor, respectively, in the antibacterial activities of afzelin.

## 1. Introduction

*P. aeruginosa* is part of a large group of free-living bacteria that are ubiquitous in the environment. It can can cause a wide range of infections and is a leading cause of illness in immunocompromised individuals. In particular, this organism can be a serious pathogen in hospitals [1]. Specifically, it can cause endocarditis, osteomyelitis, pneumonia, urinary tract infections, gastrointestinal infections, keratitis, folliculitis, ear infections, and meningitis, and is a leading cause of septicemia. *P. aeruginosa* is also a major pathogen in burn and cystic fibrosis (CF) patients and causes a high mortality rate in both of these populations [2,3]. Moreover, antibacterial resistance of *P. aeruginosa* and consumer mistrust of synthetic additives are increasing, creating a need for new antibiotics [4,5]. Therefore, natural products will likely be one of major sources of the chemical diversity needed to thwart multiple resistance mechanisms [6]. During our search for new antibacterial compounds from a taxonomically diverse plant collection, we found that *Cornus macrophylla* extract had a high antibacterial activity against *P. aeruginosa*.

*Cornus macrophylla*, or GomUiMalChae in Korean, is a dogwood with large leaves that belongs to the *Cormaceae* family and is widespread in eastern Asia. The bark extract of *C. macrophylla* has anti-cancer and immunomodulatory properties [7] as well as inhibitory activity on aldose reductase [8]. However, very little is known about other biological functions of *C. macrophylla*, especially its anti-bacterial activities. Here, the isolation of the constituents of the leaves of this plant and their antibacterial effects are described. In addition, the antibacterial compound was evaluated to provide insight into the mode of action of this compound.

## 2. Results and Discussion

### 2.1. Isolation of the Active Compound from C. macrophylla and Its Structure Determination

**Figure 1:**
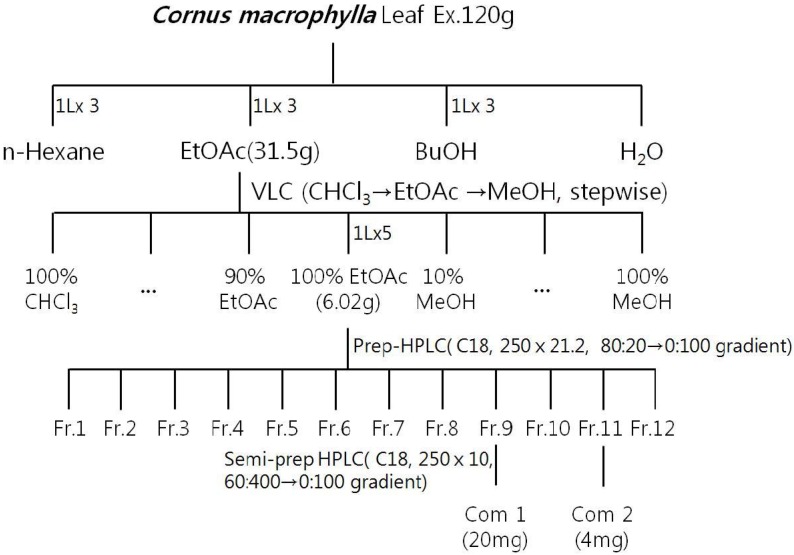
Isolation of compounds **1** and **2** from the leaves of *Cornus macrophyllla*.

**Figure 2:**
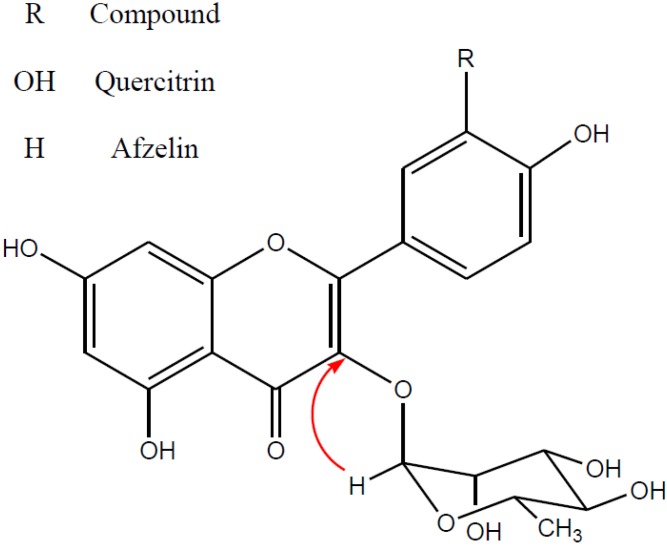
Structures of quercitrin and afzelin isolated from *Cornus macrophylla*. The arrow is the HMBC of quercitrin and afzelin.

Leaves of *C. macrophylla* (1 kg dry weight) were extracted with 80% aqueous methanol, followed by ethyl acetate, *n*-hexane, *n*-butanol or H_2_O (Figure 1). Among these extracts, the ethyl acetate extract showed the best antibacterial activities against *P. aeruginosa* (Table 1). Therefore, the ethyl acetate extract was solvent partitioned between chloroform, ethyl acetate and 80% methanol, after which the concentrated ethyl acetate soluble fraction was purified by silica gel column chromatography and subjected to preparative HPLC. Among the 12 fractions, fractions 1, 4, 6, 8, and 11 showed antibacterial activities (Table 2). In addition, fractions 8 and 11 showed stronger activities and the HPLC analysis of these fractions revealed the presence of flavonoids. Based on comparison with authentic standards, these compounds were identified as quercitrin and afzelin (Figure 2). This represents the first time afzelin was isolated from *C. macrophylla*.

To elucidate the antibacterial activity of quercitrin and afzelin isolated from *C. macrophylla* against *P. aeruginosa*, we used the disk diffusion method. Gentamycin was employed as a positive control. While quercitrin was found to have no antibacterial activity against *P. aeruginosa*, afzelin showed significant antibacterial activity; however, its activity was less potent than that of gentamycin (data not shown).

**Table 1:** Antibacterial activities of each solvent phase obtained from the extract of the leaves of *Cornus macrophylla* on the growth of *P. aeruginosa*.

Fraction	Amount (μg/disc)	Clear Zone (mm)
*n*-Hexane layer	500	N.A.
EtOAc layer	500	15
*n*-BuOH layer	500	13.1
H_2_O layer	500	N.A.
Gentamicin	10	16

N.A.: No activity.

**Table 2:** Antibacterial activity of fractions isolated by preparative HPLC against *P. aeruginosa*.

Fraction NO.	Amount (μg/disc)	Clear Zone (mm)
1	100	12.5
2	100	N.A.
3	100	N.A.
4	100	12
5	100	N.A.
6	100	12.5
7	100	N.A.
8	100	15
9	100	N.A.
10	100	N.A.
11	100	13
12	100	N.A.
Gentamicin	10	16

N.A.: No activity.

### 2.2. Antimicrobial Activity of Isolated Compound against Pseudomonas aeruginosa

The antibacterial activities of both afzelin and quercitrin were further evaluated by determining the minimum inhibitory concentration (MIC), which is the lowest concentration at which there is no growth. The MIC values of afzelin and quercitrin were determined using a two-fold serial dilution method. As shown in Table 3, while quercitrin did not inhibit the growth of *Pseudomonas aeruginosa* at any of the tested concentrations, while afzelin inhibited the growth of *Pseudomonas aeruginosa* at 31 μg/mL, indicating that the MIC of afzelin is 31 μg/mL. In addition, kaempferol, an aglycone of afzein, showed antibacterial activities and a MIC of 59 μg/mL. That is, quercitrin (afzelin with OH at C3) showed no antibacterial activity and kaempferol (afzelin without a rhamnose group) showed less potent antibacterial activity than afzellin. The MIC of gentamicin, a positive control, was also found to be 4 μg/mL. These results suggest that the hydroxyl group at C3 of the C-ring of the flavone skeleton and rhamnose group may be involved in antibacterial activity. More specifically, while the hydroxyl group at C3 of the C-ring of the flavone skeleton negatively affects antibacterial activity, the rhamnose group contributes positively to the antibacterial activity.

**Table 3:** Minimum inhibitory concentrations of quercitrin, afzelin and kaempferol.

Compound	Bacteria Tested
	MIC (μg/mL)
Quercitrin	N.A.
Afzelin	≥31
Kaempferol	≥59
Gentamicin	≥5

N.A.: No activity.

In summary, afzelin isolated from *C. macrophylla* was characterized and evaluated for its antibacterial activities against *P. aeruginosa*. We found that afzelin is one of main antibacterial compounds of *C. macrophylla* active against *P. aeruginosa*. In addition, recent studies have reported that afzelin has several properties, including anti-inflammatory, anti-tumor, and anti-apoptotic activities [9,10,11]. Taken together, the data gathered in this study suggest that afzelin may be useful as a therapeutic agent or additive for *P. aeruginosa*-related diseases. In addition, the results presented here suggest that the hydroxyl group at C3 of the C-ring of the flavone skeleton and the rhamnose group may be important groups which regulate the antibacterial activity of afzelin against *Pseudomonas aeruginosa*.

## 3. Experimental

### 3.1. General Methods

NMR spectroscopic analysis data were recorded using a JEOL JNM- LA40 spectrometer (400 MHz for ^1^H and 100 MHz for ^13^C) in CD_3_OD. The chemical shifts were reported in *δ* (ppm) units relative to the TMS signal and coupling constants (*J*) in Hz. Preparative High Pressure Liquid Chromatography (HPLC) was conducted using a Prep LC 2000 instrument and a 2487 Dual λ Absorbance detector (Waters). Silica gel (230–400 mesh, Merck) was used for column chromatography. All HPLC-grade organic solvents and bulk organic solvents were purchased from J.T. Baker (Phillipsburg, NJ, USA) and Duksan Company (Ansan, Korea).

### 3.2. Plant Material

Naturally-grown *Cornus macrophylla* (*C. macrophylla*) were collected from Jeju Island, Korea, from June to July 2007. A voucher sample was deposited at the Jeju Bio Diversity Research Institute of the Jeju Hi-Tech Industry Development Institute.

### 3.3. Extraction and Isolation

Leaves of *C. macrophylla* (1 kg dry weight) were homogenized and extracted with 80% methanol (MeOH) (10 L). The methanolic extracts (120 g) were then concentrated *in vacuo*, after which they were re-extracted with *n*-hexane (1 L × 3), ethyl acetate (EtOAc, 1 L × 3), and *n*-butanol (1 L × 3). After reducing to dryness *in vacuo*, the EtOAc fraction (31.5 g) was subjected to vacuum liquid chromatography (VLC, silica-gel, 10 × 12.6 cm), eluted with CHCl_3_-EtOAc (10:0, 9:1, 8:2, 7:3, 6:4, 5:5, 3:7, 1:9, 0:10) mixture, and then EtOAc–MeOH (4:6, 1:9, 0:10). The 100% EtOAc fraction was then purified by preparative HPLC using a leaner gradient solvent A (H_2_O) and solvent B (MeOH) for 90 min, during which time the gradient was increased from 10% solvent B to 100% solvent B in solvent A at a flow rate of 10 mL/min to give twelve fractions (Fr. 1–Fr. 12). The obtained fractions were then analyzed by HPLC and FT-NMR (^1^H, ^13^C, DEPT, COSY, HMBC, HMQC). Although HPLC fractions 8 (compound **1**) and 11 (compound **2**) were isolated, only fraction 11 (compound **2**) showed anti-microbial activity. Compounds **1** and **2** were concentrated *in vacuo* and dissolved in 10% DMSO and then used in the study. The average contents of compound **1** and compound **2** in *C. macrophylla* were 1.67% and 0.33%, respectively.

Compound **1**. Yellow powder, ^1^H-NMR (CD_3_OD) δ 7.33 (1H, d, *J* = 1.92 Hz, H-2'), 7.31 (1H, dd, *J* =1.96, 8.32 Hz, H-6'), 6.91 (1H, d, *J* = 8.32 Hz, H-5'), 6.38 (1H, d, *J* = 1.96 Hz, H-8), 6.21 (1H, d, *J* = 1.96 Hz, H-6), 5.34 (1H, d, *J* = 1.44 Hz, H-1''), 4.22 (1H, dd, *J* =1.72, 3.2 Hz, H-3''), 3.75 (1H, dd, *J* = 3.2, 9.26 Hz, H-2''), 3.42 (1H, m, H-5''), 3.35 (1H, t, *J* = 5 Hz, H-4''), 0.94 (3H, d, *J* = 6.12 Hz, H-6''), ^13^C-NMR (CD_3_OD) δ 179.81 (C-4), 166.17 (C-7), 163.37 (C-5), 159.48 (C-9), 158.7 (C-2), 149.98 (C-4'), 146.59 (C-3'), 136.37 (C-3), 123.1 (C-1'), 123.0 (C-6'), 117.07 (C-5'), 116.53 (C-2'), 106.01 (C-10), 103.7 (C-1''), 100.0 (C-6), 94.89 (C-8), 73.39 (C-4''), 72.25 (C-3''), 72.20 (C-2''), 72.05 (C-5''), 17.80 (C-6'').

Compound **2**. Yellow powder, ^1^H-NMR (CD_3_OD); δ 7.76 (2H, d, *J* = 8.76 Hz, H-2', 6'), 6.93 (2H, d, *J* = 8.88 Hz, H-3', 5'), 6.36 (1H, d, *J* = 2.2 Hz, H-8), 6.19 (2H, d, *J* = 2.44 Hz, H-6), 5.36 (1H, d, *J* = 2.2 Hz, H-1''), 4.21 (1H, dd, *J* = 1.7, 3.42 Hz, H-2''), 3.71 (H, m, H-3''), 3.60 (H, m, H-4''), 3.47 (H, m, H-5''), 0.91 (3H, d, *J* = 5.6 Hz, H-6''), ^13^C-NMR (CD_3_OD); δ 179.35 (C-4), 166.6 (C-7), 163.2 (C-5), 161.63 (C-4'), 159.24 (C-9), 158.62 (C-2), 136.15 (C-3), 131.9 (C-2', 6'), 122.62 (C-1'), 116.54 (C-3', 5'), 105.73 (C-10), 103.5 (C-1''), 100.06 (C-6), 94.92 (C-8), 73.17 (C-4''), 72.09 (C-2''), 72.04 (C-3''), 71.92 (C-5''), 17.66 (C-6'').

### 3.4. Antimicrobial Test

The antimicrobial activity of all fractions of *C. macrophylla* Leaf Ex. was evaluated using the standardized filter-paper disc-agar diffusion method, which is known as the Kirby-Bauer method [12]. *P. aeruginosa* was employed in this experiment. Bacterial cells were cultured at 37 °C in Luria-Bertani broth (BD, Difco, Franklin Lakes, NJ, USA) under aerobic conditions until their growth reached a stationary phase. The plates were then inoculated with 10^6^ CFU/mL of the test micro-organism, after which filter paper discs (8 mm, ADVANTEC, Toyo Roshi Kaicha, Ltd., Tokyo, Japan) impregnated with various concentrations of the test materials were placed on the surface of the agar plates. The plates were then incubated at 37 °C for 24 h under aerobic conditions. Antimicrobial activity was evaluated by measuring the diameter of the growth inhibition zone. Gentamycin was used as a positive control.

### 3.5. Minimum Inhibition Concentration (MIC) Test

Bacterial cells were pre-cultured for 24 h at 37 °C on an agar plate under aerobic conditions, after which the cells (10^7^) were inoculated into 2 mL Luria-Bertani broth. Subsequently, test samples were added to 2 mL Luria-Bertani broth containing the bacteria and cultured for 24 h at 37 °C under aerobic conditions. To determine the MIC of the compounds, we employed a two-fold serial dilution method [13]. The MIC value was defined as the lowest concentration that yielded no bacterial cell growth.

## 4. Conclusions

*Cornus macrophylla* showed antibacterial activity against *Pseudomonas aeruginosa*, a leading cause of illness in immunocompromised individuals. Afzelin isolated from *C. macrophylla* was found to be one of main antibacterial compounds specifically against *Pseudomonas aeruginosa*. We found that a hydroxyl group and rhamnose group may operate as a negative factor and an enhancing factor, respectively, of the antibacterial activities of afzelin.
